# Exploring the source of TYLCV resistance in *Nicotiana benthamiana*

**DOI:** 10.3389/fpls.2024.1404160

**Published:** 2024-05-28

**Authors:** Satomi Hayashi, Jacqueline M. Souvan, Julia Bally, Felipe F. de Felippes, Peter M. Waterhouse

**Affiliations:** ^1^ Centre for Agriculture and the Bioeconomy, Queensland University of Technology, Brisbane, QLD, Australia; ^2^ Australian Research Council Centre of Excellence for Plant Success in Nature and Agriculture, Queensland University of Technology, Brisbane, QLD, Australia

**Keywords:** *Nicotiana benthamiana*, tomato yellow leaf curl virus, disease resistance, RNA-dependent RNA polymerase, Ty genes

## Abstract

Tomato Yellow Leaf Curl Virus (TYLCV) is one of the most devastating pathogens of tomato, worldwide. It is vectored by the globally prevalent whitefly, Bemisia tabaci, and is asymptomatic in a wide range of plant species that act as a virus reservoir. The most successful crop protection for tomato in the field has been from resistance genes, of which five loci have been introgressed fromwild relatives. Of these, the Ty-1/Ty-3 locus, which encodes an RNA-dependent RNA polymerase 3 (RDR3), has been the most effective. Nevertheless, several TYLCV strains that break this resistance are beginning to emerge, increasing the need for new sources of resistance. Here we use segregation analysis and CRISPR-mediated gene dysfunctionalisation to dissect the differential response of two isolates of Nicotiana benthamiana to TYLCV infection. Our study indicates the presence of a novel non-RDR3, but yet to be identified, TYLCV resistance gene in a wild accession of *N. benthamiana*. This gene has the potential to be incorporated into tomatoes.

## 1 Introduction

Their rapid life cycles, small genomes, short stature, and amenability to genetic transformation have made *Arabidopsis thaliana* and *Brachypodium distachyon* model research plants. The laboratory isolate (LAB) of *Nicotiana benthamiana* has a larger stature and a much more complex genome than these species, but it has been extensively used as a platform to study protein-protein interactions, functional genomics, gene silencing and bioproduction of therapeutics and vaccines (Bally et al., 2018, 2015). With its recently assembled genome sequence (Ranawaka et al., 2023; Ranawaka et al., 2023), amenability to transient and stable genetic transformation, and an ever-increasing prevalence in research publications, the plant probably merits classification as a model species. In addition to the LAB isolates distributed around the world, which seem to have come from the seed of a single plant collected from Central Australia, five wild accessions of *N. benthamiana* have been collected from geographically distinct regions of Australia, named accordingly, and their genomes sequenced (Bally et al., 2015; Ranawaka et al., 2023). Apart from one exception, the accessions are morphologically and physiologically distinct from each other, and from LAB. This not only provides insights about the diversity within the species but also broadens available genetic resources. One of the key differences between LAB and wild accessions is virus susceptibility. When challenged with a number of viruses, almost all of the wild accessions were much more resistant than LAB (Bally et al., 2015). All but one of the viruses employed an RNA genome, and the pronounced susceptibility of LAB was attributed to its lack of a functional RNA-dependent RNA polymerase 1 (RDR1), a key player in antiviral RNAi-mediated defense in plants (Xie et al., 2001; Yang et al., 2004; Kumari et al., 2021; For a comprehensive review on the role of small RNA for viral immunity, refer to Lopez-Gomollon and Baulcombe (2022)). However, LAB also showed increased susceptibility to Tomato yellow leaf curl virus (TYLCV; Bally et al., 2015), a DNA virus belonging to the Begomovirus genus in the *Geminiviridae*. This raised at least three possibilities: (1) RDR1 aids the production of small RNAs which guide the RNAi pathway to cleave TYLCV mRNAs or mRNAs of endogenous genes required for TYLCV infection, (2) RDR1 aids the production of small RNAs which guide the RNAi pathway to suppress TYLCV by methylating its genomic DNA, and (3) there is a non-RDR1 TYLCV resistance gene in the wild accessions, but not in LAB.

TYLCV produces mild disease in most Solanaceous crops but causes severe symptoms and drastic yield losses in tomato (Polston et al., 2006; Butterbach et al., 2014). It has a wide host range and is vectored, in temperate regions of the world, by the prevalent whitefly, *Bemisia tabaci.* Controlling the disease by mechanical or chemical treatments has been difficult (Horowitz et al., 2007), so the tomato industry relies heavily on resistance genes introgressed from wild relatives. This makes new sources of resistance highly desirable. To date, six quantitative trait loci (QTL) for TYLCV resistance, namely *Ty-1* to *Ty-6*, have been isolated from wild accessions of tomatoes. *Ty-1* and its allelic variant *Ty-3* are RDRs from the γ-clade (Verlaan et al., 2013) and are currently the main sources of resistance in breeding programs. *Ty-2* is a nucleotide-binding domain and leucine-rich repeat containing (NLR) protein, and *ty-5* is a messenger RNA surveillance factor (Pelota) (Lapidot et al., 2015; Yamaguchi et al., 2018). Although *Ty-4* and *Ty-6* loci have been genetically mapped to chromosome regions in the tomato genome (Ji et al., 2009; Gill et al., 2019), neither the genes responsible nor their mode(s) of action, have been elucidated.

Here, we investigate the source and nature of TYLCV resistance in *N. benthamiana.*

## 2 Materials and methods

### 2.1 Plant material and TYLCV infection

The two *N. benthamiana* accessions used in this study, LAB and QLD, have been described previously in Bally et al. (2015). Plants were grown in a UQ23 soil mix (30% coco peat, 70% composted pine bark) supplemented with Osmocote slow-release fertiliser (5 ml/L soil) under controlled environment (25 °C, 16/8 h light/dark condition).

The LAB x QLD F1S1 population was obtained by first creating an F1 individual from the LAB and QLD parent plants. This was achieved by emasculating the flower of QLD, followed by pollination using a flower from the LAB isolate. The resulting F1 individual was allowed to self-pollinate and the seeds collected.

Inoculation of plants with TYLCV and Tomato yellow dwarf virus (TYDV) was conducted on three-weeks old plants using the Agroinfiltration method (de Felippes and Weigel, 2010). The *Agrobacterium tumefaciens* (strain GV3101) carrying an infectious clone of TYLCV (GenBank GU178815) or TYDV (Kato et al., 2020) at final concentration of OD_600 = _0.1 (in infiltration buffer: MES (20 mM), MgCl_2_ (5 mM) and acetosyringone (100 µM)) were used as an inoculum. The TMV-U1 was inoculated mechanically by macerating infected leaf material in 100 mM phosphate buffer and rubbing it on a lower leaf lightly dusted with carborundum.

### 2.2 Quantification of viral load in systemic tissue

For performing absolute quantification of the viral DNA, a standard curve was first created using the plasmid carrying the infectious clone in a serial dilution (1 to 10^8^ copies). The qPCR was performed using the CFX384 system (BioRad) with GoTaq^®^ qPCR Master Mix (Promega) and TYLCV specific primers (forward: 5’-CAACGGTTCTTCGACCTGGT-3’ and reverse: 5’-TGCTGACCTCCTCTAGCTGAT-3’, 0.4 µM each). Total DNA (30 ng) extracted from the apical leaf tissue at 3 weeks post inoculation (wpi) was used as a template for measuring the level of systemic infection.

### 2.3 Bioinformatic analysis

The sequence analysis was performed using Geneious Prime software (Biomatters). All sequences for *N. benthamiana* LAB and QLD were extracted from the *N. benthamiana* genome resource available on WebApollo Browser (www.apollo.nbenth.com; Ranawaka et al., 2023). Orthologs for *Ty*-genes were identified using the inbuilt BLAST function on the *N. benthamiana* genome using the tomato protein sequence as the query. The phylogenetic tree for the *RDR* genes was created with Neighbour-Joining method using a mRNA sequence without the 5’ and 3’ UTRs (to account for genes with the presence of early stop codons).

### 2.4 Creation of CRISPR-Cas9 constructs

All CRISPR-Cas9 constructs were generated using pCas9 plasmid described by (Naim et al., 2020). For each *RDR* gene, two target sites were identified either with WU-CRISPR software (Wong et al., 2015; for *NbRDR1*) or CRISPR-P 2.0 tools (Liu et al., 2017; for *NbRDR3* and *NbRDR5*). The two target sites were incorporated into the tRNA-gRNA cassette as described in Xie et al. (2015) via golden gate method and cloned into the pCas9 plasmid. Primers used for the assembly of the constructs are provided in **Supplementary Table 2**. The resulting CRISPR-Cas9 constructs were confirmed by Sanger sequencing before being transformed into *A. tumefaciens* strain GV3101. The cutting efficiency of the constructs was assessed by transient expression analysis (dropout assay for the CRISPR-Cas9 target region) as previously described in Naim et al. (2020), using primers listed in **Supplementary Table 2**. Constructs that showed signs of dropouts, demonstrating efficient targeting by CRISPR-Cas9 at both target sites, were taken further for *Agrobacterium*-mediated transformation.

### 2.5 Generation of *N. benthamiana* RDR knockout mutants

Four to five weeks old *N. benthamiana* plants were subjected to *Agrobacterium* mediated transformation as described in Naim et al. (2020). Resulting shoots with root growth under appropriate antibiotic selection were tested for editing at the target sites. For this, a small piece of leaf tissue (approx. 5 mm in diameter) were collected for a Rapid Release DNA extraction (Thomson and Henry, 1995). Genotype PCR was performed using 2x 2G Robust HotStart Ready Mix (KAPA Biosystems) and gene specific primers for dropout analysis (**Supplementary Table 2**). PCR products were purified using SureClean Plus (Bioline) following the manufacturer’s instructions, and sequenced at Macrogen Inc. (Seoul, Korea) using one of the PCR primers. Individual plants at T_0_ plants, identified as having editing at the target site (in a heterozygous state), were allowed to self-fertilise to produce the T_1_ generation. The T_1_ individuals having homozygous edits were identified by PCR genotyping, and T_2_ plants resulting from these individuals were used for further phenotype analysis.

For the generation of the double *rdr* mutants, single homozygous *rdr* mutant plants were crossed to make heterozygous double *rdr* mutants (F1). The F1 plants were allowed to self-fertilise, and the F2 individuals were screened for homozygous edits for the two target *RDR* genes. The F3 homozygous double *rdr* mutant plants were taken for further phenotype analysis.

### 2.6 Genotyping for TYLCV resistant candidate genes on the LAB x QLD F1S1 individuals

The 24 LAB x QLD F1S1 individuals were challenged with TYLCV, their resistant phenotype recorded, and their DNA extracted. Total DNA (20 ng) was used for genotyping analysis for RDR1, RDR3 and the Pelota b genes.

For the RDR1 gene, a primer pair, spanning the 72 bp insertion in the LAB RDR1 (Bally et al., 2015), which binds to both LAB and QLD genes was designed (**Supplementary Table 2**), to give product sizes of 347 bp and 275 bp for NbLRDR1 and NbQRDR1, respectively. For the RDR3 and Pelota b genes, single nucleotide polymorphism (SNP) between the LAB and QLD sequence, which dictate presence or absence of the restriction site were first identified by sequence analysis (i.e., presence of *Hpa*II and *Scr*FI recognition sites for NbLRDR3 and NbQPelota b, respectively, while absent in the other counterpart). These SNPs were then used as markers for restriction fragment length polymorphism (RFLP) studies, where PCR products spanning the SNPs were subjected to restriction enzyme digestion. The primer sequences used for this experiment are provided in **Supplementary Table 2**.

## 3 Results

### 3.1 *N. benthamiana* LAB and QLD show differential susceptibility to TYLCV infection

**Figure 1:**
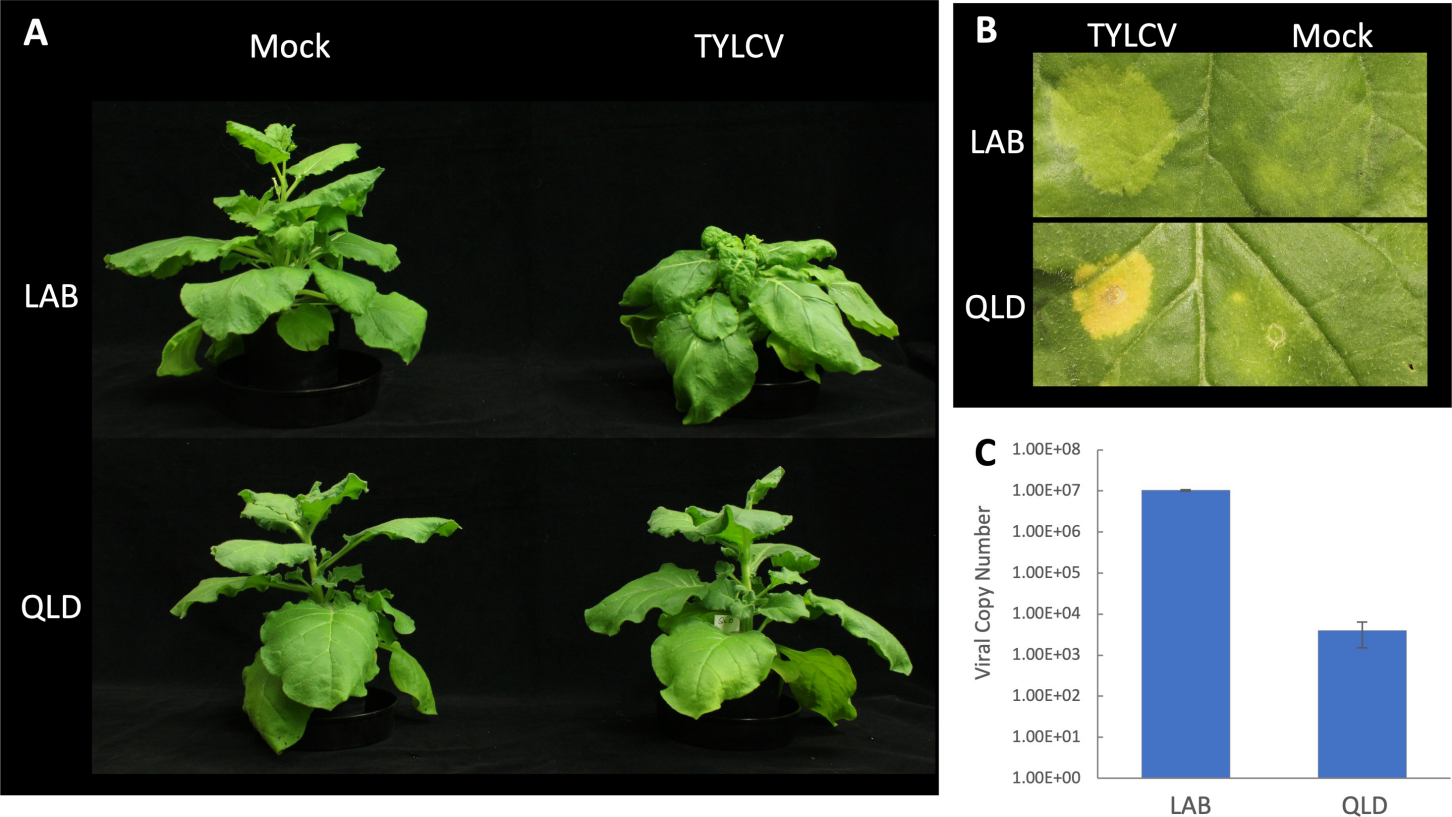
*N. benthamiana* LAB and QLD accessions displaying differences in susceptibility to TYLCV. **(A)** LAB and QLD plants at 3 weeks post Agroinfiltration with the *Agrobacterium* either carrying the TYLCV infectious clone (TYLCV), or no-vector control (Mock); **(B)** Development of chlorotic leaf patch at the site of infiltration after 5 dpi; **(C)** Copy number of viral genome detected by qPCR in the systemic tissue at 3 wpi (n=3).

Three-week-old plants of LAB and a wild isolate from Queensland (QLD) were Agro-inoculated with an infectious clone of TYLCV. This resulted in pronounced chlorosis at the infiltration sites 5 days post infiltration (dpi), especially in QLD, and systemic disease symptoms were apparent in the young leaves of LAB 10 dpi (**Figure 1**). Over the subsequent weeks, the LAB plants developed stunting, leaf crinkling, and flower deformation. In contrast, the inoculated QLD plants showed no obvious disease symptoms. Nevertheless, TYLCV was detectable in the apical tissue of QLD plants at 3 wpi, albeit at a 10,000-fold lower levels than in LAB (**Figure 1C**).

### 3.2 TYLCV resistance in QLD accession is governed by a single dominant locus

**Figure 2:**
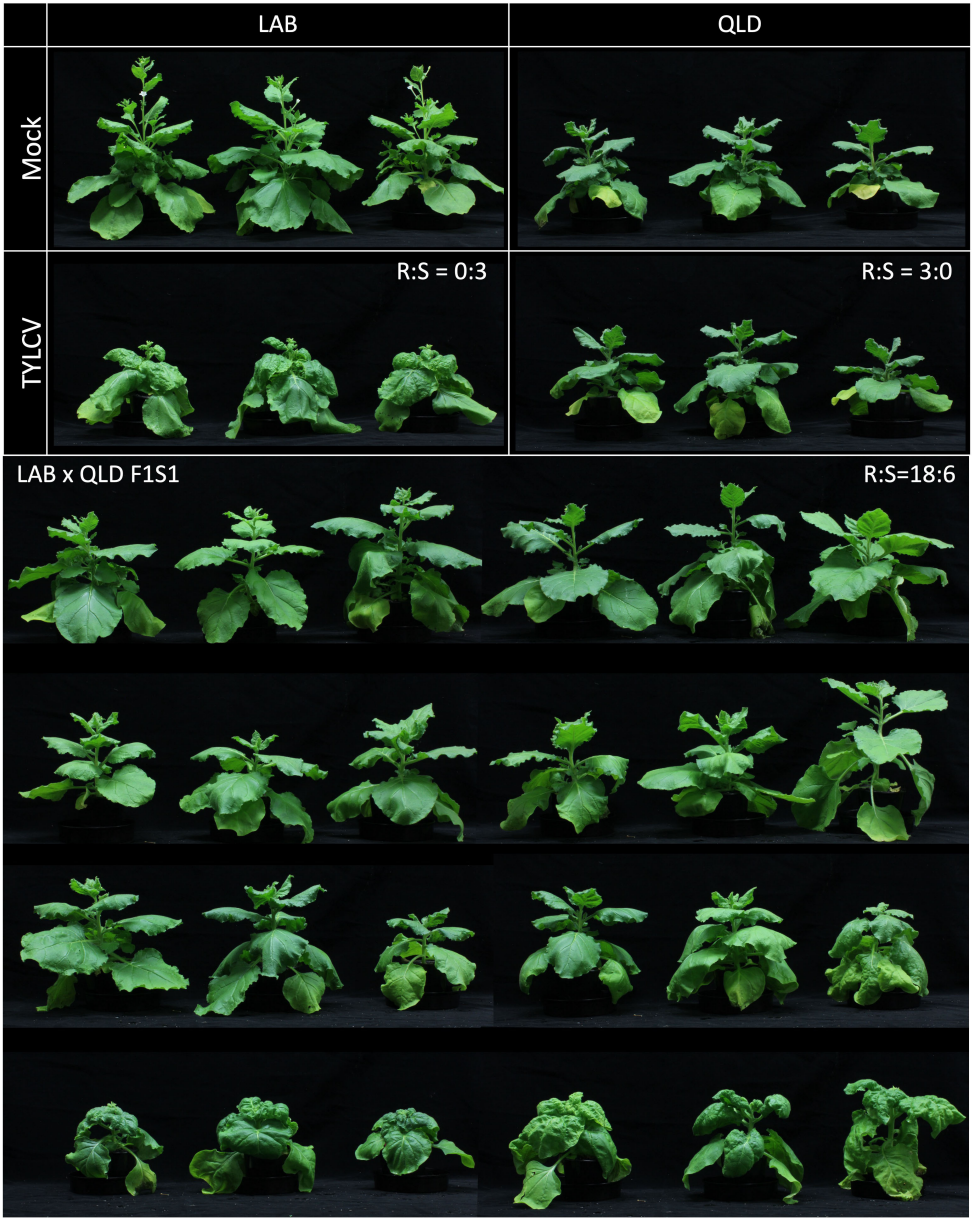
Segregation of TYLCV resistant phenotype in the F1S1 population of *N. benthamiana* LAB and QLD ecotype. The F1S1 individuals from LAB and QLD cross (n=24) was assessed for TYLCV resistance at 3 wpi. In this population, 18 plants (top 3 rows of plants) showed resistant phenotype (R) whereas 6 plants (bottom row) displayed TYLCV susceptible phenotype (S).

To better understand the genetics of the TYLCV resistance in QLD, a population derived from a cross between LAB and QLD was examined. Twenty progeny plants (F1) from the LAB x QLD cross were Agro-inoculated with the infectious TYLCV clone. None of the plants showed TYLCV symptoms, suggesting that a dominant resistance gene (or genes) was inherited from the QLD parent. To examine this further, an F1 line was allowed to self-fertilise and 24 of the F1S1 progeny were challenged with TYLCV. Six of these developed severe disease symptoms and the remaining 18 appeared disease-free (**Figure 2**). No clear intermediate phenotypes were observed. This 3:1 ratio for resistant to susceptible phenotype strongly suggested that there is a single dominant gene that is responsible for the TYLCV resistance in QLD, and it is being inherited in a simple Mendelian fashion.

### 3.3 The *N. benthamiana* LAB isolate lacks a full-length *Ty-1*/*Ty-3*-like gene

**Figure 3:**
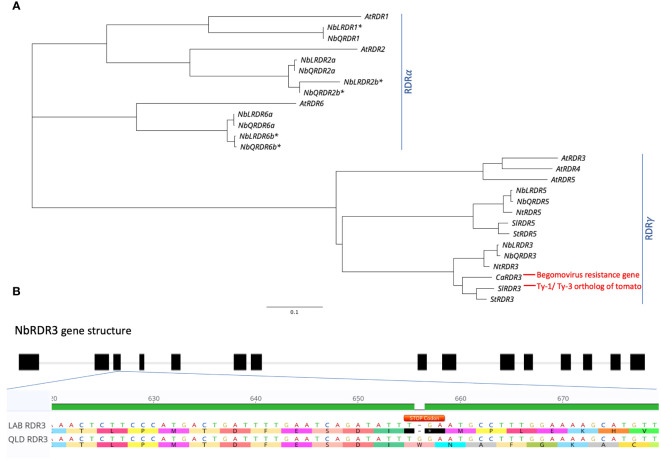
Identification of *Ty-1*/*Ty-3* ortholog in *N. benthamiana* LAB and QLD genomes. **(A)** Phylogenetic tree of *Ty-1/Ty-3* orthologs in number of different plant species including *Arabidopsis thaliana* (At), tomato (Sl), potato (St), pepper (Ca), tobacco (Nt) and *N. benthamiana* (Nb), as well as other *RDR* genes (*RDR1*, *RDR2* and *RDR6*) of *N. benthamiana* and *A. thaliana*. **(B)** Identification of early stop codon in the coding sequence of RDR3 in LAB isolate. A deletion of a base contributing to an early introduction of a stop codon (TGA; annotated in Orange) was found on the third exon of the LAB RDR3, which is absent in the RDR3 of QLD ecotype. Accession for each gene is provided in **Supplementary Table 1**. The Asterisk (*) Denotes confirmed unfunctional/truncated gene in *N. benthamiana*.

To investigate whether the TYLCV resistance in QLD is governed by a *Ty-1/Ty-3*-like gene, the genome sequences of LAB and QLD were BLAST-searched for orthologs of *Solanum chilense Ty-1/Ty-3*, an *Arabidopsis* RDR3/4/5 homolog (Verlaan et al., 2013). In each genome, two loci, already annotated as either RDR3 or RDR5, were identified: one on chromosome 10 (NbLab360C10:7750874-7763631 and NbQld183C10:7520886-7533697) and the other on chromosome 12 (NbLab360C12:13708941-13719463 and NbQld183C12:13404624-13415384). These putative RDRs fall into the RDRγ-type, along with the tomato *Ty-1/Ty-3* orthologs, SlRDR3 (previously known as Solyc06g051170, Solyc06g051180 and Solyc06g051190 (ITAG2.3 version); Verlaan et al., 2013) and CaRDR3, which has been identified as the *Begomovirus* resistance gene in pepper (Koeda et al., 2022). *N. benthamina* is a rapidly diploidising allotetraploid with both homoeologs being present for approximately half of its genes (Ranawaka et al., 2023). However, while both homoeologs of *NbRDR2* and *NbRDR6* (which along with *NbRDR1* cluster within the RDRα type) are detectable, only one copy of *NbRDR3* and one copy of *NbRDR5* appear to have been retained (**Figure 3A**). Further analysis revealed that there is a stop codon (at position 657 bp) in the third exon of LAB *NbRDR3* (**Figure 3B**), which is absent in QLD. This prematurely terminates the gene’s open reading frame to encode a 218 aa protein. Functional RDR3 genes in plants encode ~1000 aa proteins, suggesting that NbRDR3 in QLD might act like *Ty-1*/*Ty-3* in tomato and provide resistance against TYLCV.

### 3.4 Mutating *NbRDR3* in QLD does not abolish its TYLCV resistance

**Figure 4:**
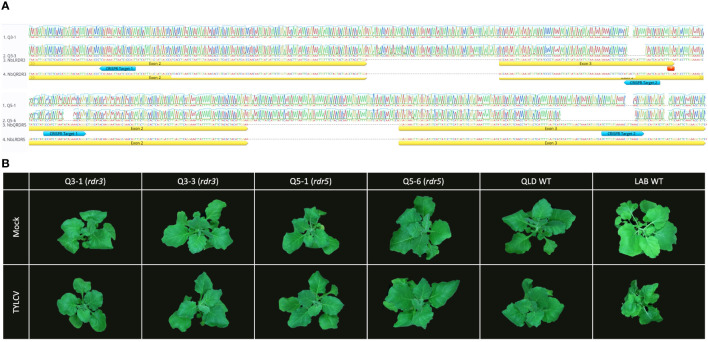
Mutation of *RDR3* or *RDR5* genes in QLD has no effect on TYLCV resistance. **(A)** Sequence of the CRISPR-Cas9 induced *RDR3* and *RDR5* mutant lines (*rdr3*: Q3-1 and Q3-3, and *rdr5*: Q5-1 and Q5-6) with edits on the two target sites (blue arrow). **(B)** The QLD *rdr3* and *rdr5* plants, showed no disease symptom upon TYLCV inoculation, whereas LAB WT plant displaying the typical TYLCV infection symptoms at 3 wpi.

To test whether the *NbRDR3* allele in QLD is providing TYLCV resistance, the gene was mutated in QLD using CRISPR-Cas9 technology. Sequencing a library of plants transformed with CRISPR-Cas9 constructs, targeted to cleave a site in *NbRDR3*, lead to the identification of two mutant lines, *rdr3-*Q3-1 and *rdr3-*Q3-3. These had 2 nt and 10 nt deletions, respectively (at position 630-650 bp; **Figure 4A**). The deletions caused frame shifting and early termination of the coding sequence, resembling the situation in LAB *NbRDR3*. The plants had no obvious developmental or morphological defects and were visually indistinguishable from wild-type QLD plants but unexpectedly, when challenged with TYLCV showed no signs of susceptibility (**Figure 4B**).

RDR5 and RDR3 have similar sequences and might be expected to have similar functions. Thus, a functional NbRDR5 could be responsible for the TYLCV resistance in QLD. To test this, *NbRDR5* knockout lines were generated in QLD using CRISPR-Cas9. Mutant lines, *rdr5-*Q5-1 and *rdr5-*Q5-6 had a 4 nt (position 771-775) and a 40 nt (position 737-776) deletion, respectively, but, like the *NbRDR3* knockout lines, they retained resistance to TYLCV (**Figure 4**).

To eliminate the possibility that the resistance of the single gene mutants was due to the compensatory effect of the other active RDRγ family member, double knockout lines (*rdr3/rdr5*) were created and challenged with TYLCV. These homozygous *rdr3/rdr5* lines also showed no significant developmental differences nor increased susceptibility to the virus.

### 3.5 Susceptibility of LAB to TYLCV is not due to the loss of function of the *RDR1*

RDR1 is known to play a role in viral resistance by producing virus-derived small interfering RNAs (vsiRNA) and viral-activated siRNA (vasiRNA) (Wang et al., 2010; Cao et al., 2014). Indeed, LAB’s hyper-susceptibility to a wide range of viruses has been attributed to its lack of a functional RDR1. Therefore, we investigated whether RDR1 might play a role in QLD’s TYLCV resistance.

**Figure 5:**
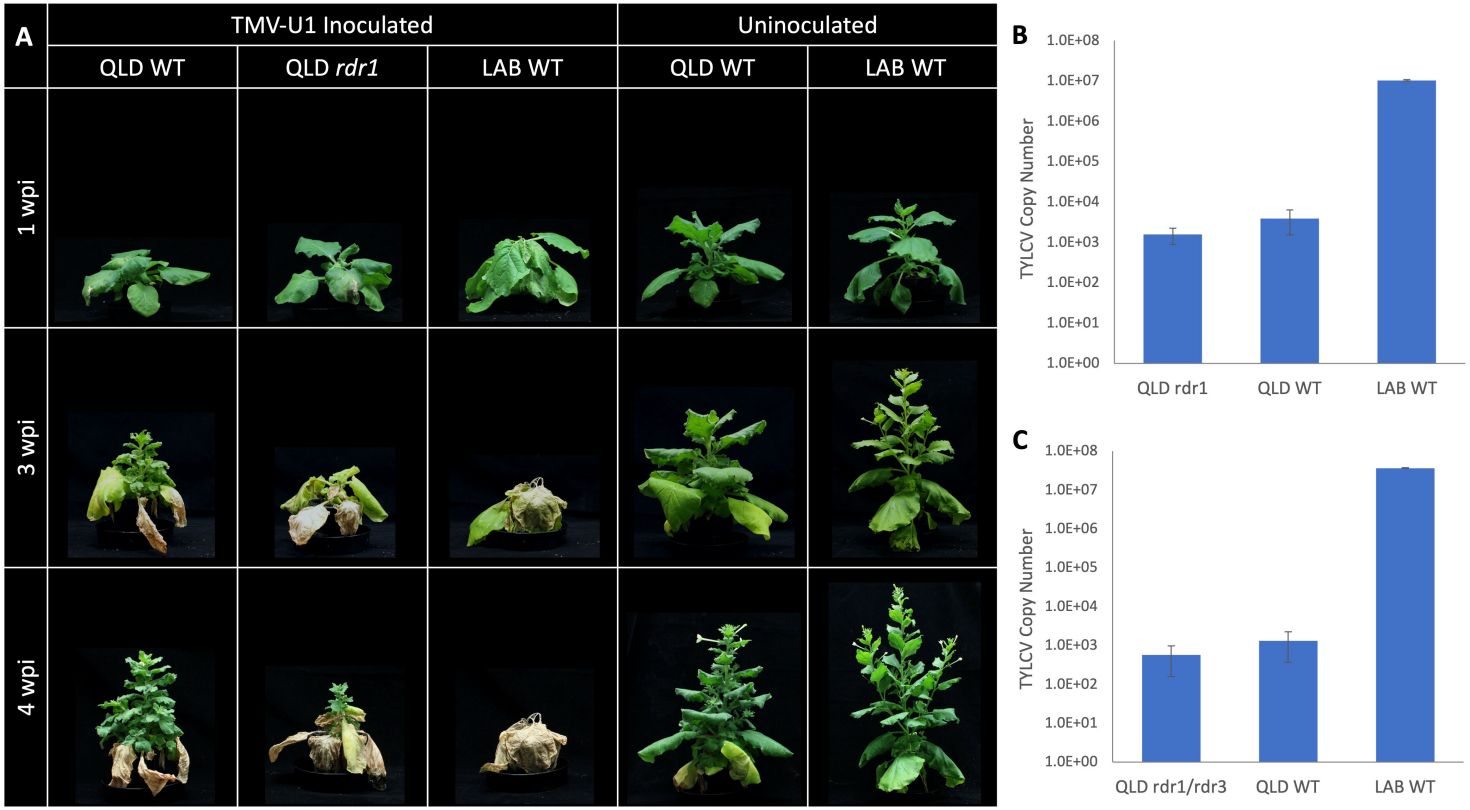
TMV and TYLCV challenge of the QLD *RDR1* knockout mutant. **(A)** Disease symptoms observed at 3 different timepoints in LAB and QLD plants after TMV inoculation. LAB WT and QLD *rdr1* show more pronounced disease symptoms than QLD WT. **(B)** Systemic accumulation of TYLCV in QLD *rdr1 mutant* and respective WT plants at 3 wpi (n=6 for the *rdr1* mutant (3 plants each from the two independent lines), and n=3 for each wild-type plants.). **(C)** Accumulation of TYLCV in the apical tissue of *rdr1/rdr3* double mutant of QLD and respective WT plants at 3 wpi (n=3, for each line).

There is 99.9% sequence identity between the coding sequence of *NbLRDR1* and *NbQRDR1*, except for a 72 bp insertion in the middle of the gene. Two CRISPR-Cas9 knockout mutant lines, *rdr1-*Q1-1 and *rdr1-*Q1-3, with 5 and 4 bp deletions, respectively, were generated in QLD and challenged with Tobacco Mosaic Virus (TMV), whose infection is known to be affected by the function of *RDR1* (Xie et al., 2001; Yang et al., 2004; Qin et al., 2017). Three weeks after inoculation, both LAB and the QLD *Nbrdr1* mutant lines showed more pronounced stunting, developmental defects, and leaf necrosis than the wild-type QLD (**Figure 5A**), confirming the expected protective role of RDR1. The *Nbrdr1* QLD mutants were also challenged with TYLCV, however, there was no increase in susceptibility nor virus titre (**Figure 5B**) when compared to inoculated wild-type QLD plants. As with the single mutants, the double *rdr1*/*rdr3* QLD mutant (created by crossing *rdr1-*Q1-1 and *rdr3-*Q3-3) showed no sign of increased TYLCV susceptibility or significantly increased viral titre (**Figure 5C**).

### 3.6 Searching for the TYLCV resistance gene in QLD by sequence analysis

**Figure 6:**
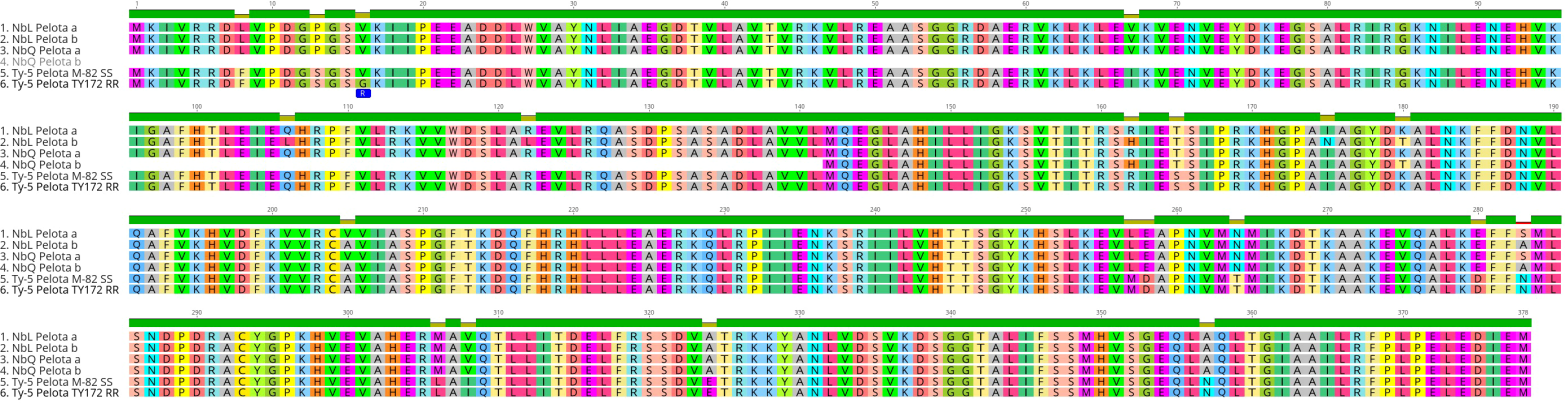
Sequence alignment of Pelota proteins in *N. benthamiana* and tomato. Sequence analysis identified 2 copies of a Pelota ortholog in both LAB and QLD, with one gene in QLD, *NbQPelota b*, which is missing the first 141 amino acid. The 16^th^ amino acid (blue) is responsible for the resistance in tomato line TY172 (Glycine^16^), whereas all full-length *N. benthamiana* Pelota orthologs have Valine^16^, which is found in the susceptible tomato line, M82.

Our results show that TYLCV resistance is not conferred by the *Ty-1/Ty-3*-like *NbRDR3* or *NbRDR5* genes in QLD. Therefore, we investigated whether there are orthologs of the other tomato *Ty* genes in the QLD *N. benthamiana* genome (**Table 1**). Using the *ty-5* (Pelota) protein sequence as a BLAST query, we identified two Pelota-likes genes located in homoeologous chromosomes (chromosome 4 and 14) in both, LAB and QLD genomes (*NbLPelota a*; NbLab360C04:9012710-9023205, *NbLPelota b*; NbLab360C14:67088265-67097654*, NbQPelota a*; NbQld183C04:9114659-9125745, *NbQPelota b*; NbQld183C14:66275667-66282816) (**Figure 6**). Both copies of the Pelota ortholog encode full-length proteins in LAB, whereas only one copy encodes a full-length protein in QLD (*NbQPelota a*). *NbQPelota b* lacks the 5’ end of the coding region (missing Exon 1 to 3 coding for the first 141 aa), where the gene is disrupted by the insertion of transposable elements. *NbPelota a* from LAB and QLD encode identical proteins and all full-length Pelota-like proteins in *N. benthamiana* have a Valine in position 16, which is the landmark for the TYLCV susceptibility allele in tomato (Lapidot et al., 2015). Furthermore, Pelota, with a Glycine in this position, gives resistance in the recessive state in tomato and pepper against TYLCV and pepper-infecting Begomovirus (Lapidot et al., 2015; Koeda et al., 2021), whereas the TYLCV resistance in QLD is a dominant trait. Given the above observations, these Pelota orthologs are unlikely to be conferring TYLCV resistance in QLD.

**Table 1:** TYLCV resistance locus (*Ty*) and their *N. benthamiana* orthologs.

QTL	Source	Gene	Reference	*N. benthamiana* orthologs
*Ty-1*/*Ty-3*	*Solanum chilense*	RNA dependednt RNA polymerase (RDR) of the RDRY clade	Verlaan et al. (2013); Butterbach et al. (2014)	NbLab360C12:13708941-13719463; NbQld183C12:13404624-13415384
*Ty-2*	*Solanum habrochaites*	Nucleotide-binding leucine-rich repeat (NLR) protein	Yamaguchi et al. (2018)	No highly similar gene found
*Ty-4*	*S. chilense*	Unknown	Kadirvel et al. (2013)	NA
*ty-5*	*S. lycopersicum* cultivar Tyking/*Solanum habrochaite*	Messenger RNA surveillance factor, Pelota	Anbinder et al. (2009); Lapidot et al. (2015)	NbLab360C04:9012710-9023205; NbLab360C14:67088265-67097654; NbQld183C04:9114659-9125745; NbQld183C14:66275667-66282816*
*Ty-6*	*S. chilense*	Unknown	Gill et al. (2019)	NA

The asterisks (*) denotes incomplete protein.

A tBLASTn search for the ortholog of tomato *Ty-2*, an NLR protein (TYNBS1), retrieved many NLR-like protein-coding loci from both LAB and QLD genomes. However, none had the level of similarity expected for an orthologous gene. For example, the most similar loci in *N. benthamiana* to TYNBS1 were NbLab360C06:144295701-144308511 and NbQld183C16:154873464-154880640, which shared < 60% identity at amino acid level with the query sequence, whereas classical orthologous genes share much higher identity, as seen for the Pelota orthologs which share approximately 95% identity.

### 3.7 Genotyping studies confirm the lack of participation of RDR1, RDR3 and Pelota b in TYLCV resistance

To further verify that the forms of RDR1, RDR3 or Pelota b in QLD do not confer resistance to TYLCV, we conducted a genotyping study on the 24 F1S1 (LAB x QLD) individuals that had been used to identify the inheritance pattern for TYLCV resistance (**Figure 2**). For RDR3 and Pelota b genes, we utilised SNPs for RFLP analysis, and for RDR1 we used the presence or absence of a 72 bp insertion (present in LAB and absent in QLD).

The results showed no correlation between the allelic make up for the candidate genes with the different TYLCV infection phenotypes of the F1S1 individuals (**Table 2**; **Supplementary Figure 1**). More importantly, for each of the candidate genes, at least one individual within the 6 susceptible lines was identified as homozygous for the QLD allele. Taking into account that TYLCV resistance in this population is thought to be driven by a dominant allele originating from QLD, these results underpin the conclusion that that neither RDR1, nor RDR3 nor Pelota b from QLD is responsible for the TYLCV resistance.

**Table 2:** TYLCV infection phenotype and genotype for the resistance gene candidates in the LAB x QLD F1S1 indivisuals.

Plant ID	Phenotype	Genotype		
		RDR1	RDR3	Pelota b
1	R	Het	LAB	Het
2	R	Het	Het	Het
3	R	Het	LAB	Het
4	R	Het	Het	Het
5	R	Het	LAB	LAB
6	R	QLD	Het	Het
7	R	QLD	Het	Het
8	R	QLD	LAB	LAB
9	R	QLD	Het	Het
10	R	QLD	LAB	Het
11	R	QLD	Het	Het
12	R	Het	QLD	Het
13	R	Het	LAB	Het
14	R	QLD	Het	Het
15	R	QLD	LAB	LAB
16	R	Het	Het	Het
17	R	QLD	LAB	Het
18	R	QLD	Het	LAB
19	S	Het	LAB	QLD
20	S	QLD	LAB	QLD
21	S	Het	Het	QLD
22	S	Het	QLD	LAB (?)
23	S	QLD	Het	Het
24	S	QLD	LAB	Het

R, resistant; S, susceptible; LAB, homozygous for LAB allele; QLD, homozygous for QLD allele; Het, heterozygous. Pink and blue colour represent phenotype or genotype attributed to that of QLD and LAB, respectively.

### 3.8 *N. benthamiana* TYLCV resistance gene does not protect against TYDV infection

**Figure 7:**
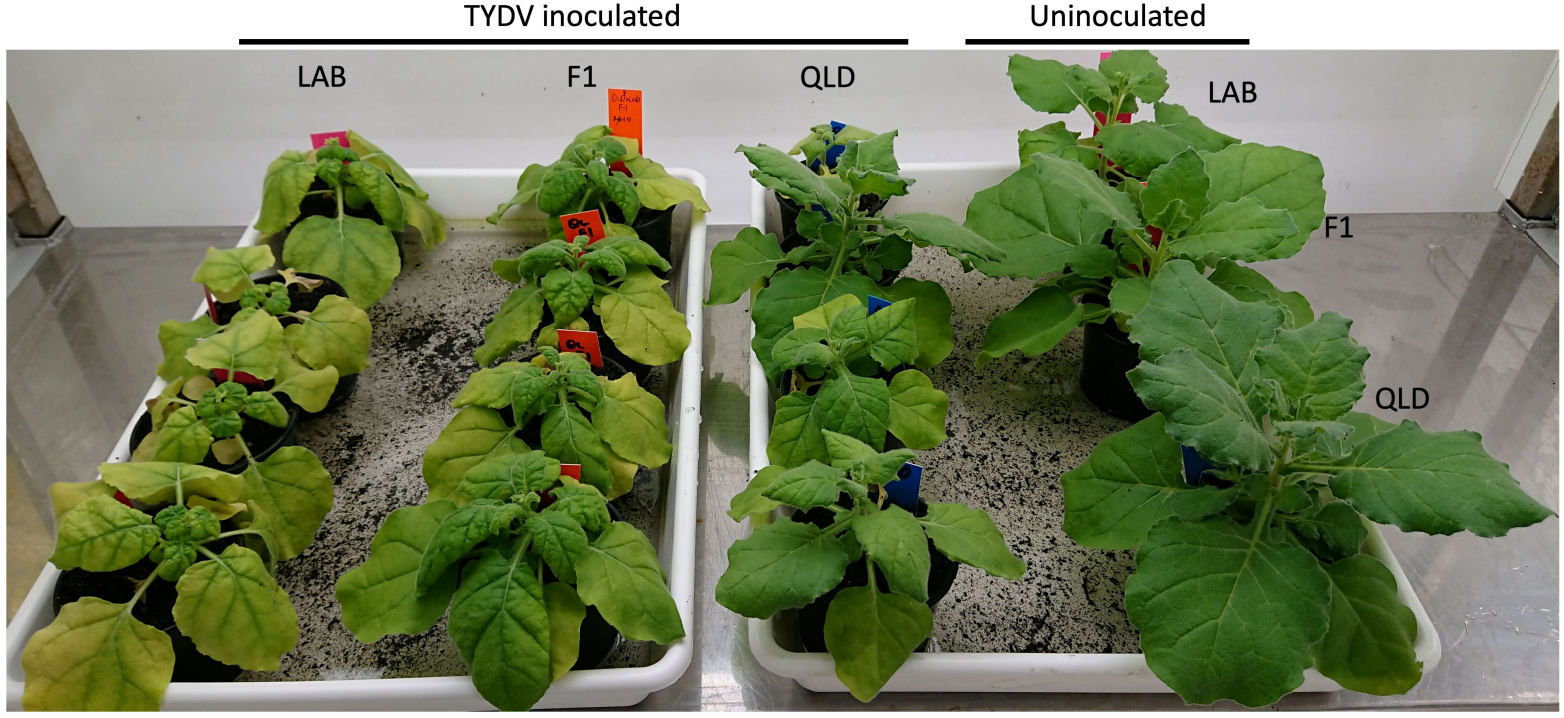
Susceptibility to TYDV in *N. benthamiana* LAB, QLD and its F1 progeny. Plants at 3 weeks post Agroinfiltration with TYDV infectious clone (from left: LAB, F1 and QLD plants) and uninoculated control (most right; one plant from each genotype; from top: LAB, F1 and QLD).

Tomato yellow dwarf virus (TYDV) is a mastrevirus in the *Geminiviridae* family. Like TYLCV, the genome of the TYDV is comprised of a single circular ssDNA molecule. To better understand the mode and breadth of the TYLCV resistance in QLD, both LAB and QLD plants, and their F1 progeny were challenged with TYDV (**Figure 7**). In contrast to the responses to TYLCV, both accession and the F1 progeny were highly susceptible to TYDV infection, showing severe stunting and chlorosis. Among the three genotypes, LAB was the most affected, followed by the F1 plants. This result suggests that the TYLCV resistance in QLD accession is not effective in suppressing the disease imposed by TYDV, and that the action of the resistance gene is likely specific to TYLCV.

## 4 Discussion

Tomato yellow leaf curl disease, of which TYLCV is the major causal pathogen, is the most destructive disease in tomato fields (Lefeuvre et al., 2010). The best control options to manage the disease are good cropping practices and the use of resistant varieties (Mahmoud, 2015; Dhaliwal et al., 2020; Prasad et al., 2020). However, TYLCV has a rapid mutation rate (Duffy and Holmes, 2008) and the continuous use of a limited number of resistance genes enhances the risk of emerging resistance-breaking virus strains. Unsurprisingly, there are already reports of *Ty-2* and *Ty-1* resistance-breaking viruses (Belabess et al., 2015; Ohnishi et al., 2016; Panno et al., 2018; Granier et al., 2019). Therefore, approaches such as gene pyramiding and identification and application of new resistance sources, are key to minimising the development of such strains and avoiding serious disease outbreaks in the field.

In this study, we investigated a source of TYLCV resistance in a wild isolate of *N. benthamiana*. The LAB isolate of *N. benthamiana* has long been utilised as a model species to study plant-virus interactions, which is enabled by its hyper-susceptibility to range of viruses. In addition, its amenability to *Agrobacterium* mediated transformation (transiently via Agoinfiltration, and stably through tissue culture) and virus induced gene silencing (VIGS) makes it a perfect platform for characterising gene functions, including those of pathogen resistance genes and the underlying mechanisms of disease resistance. Our study has shown that *N. benthamiana* QLD is highly resistant and nearly immune to TYLCV infection. A chlorotic patch develops at the site of TYLCV Agroinfiltration, resembling a hypersensitive response (HR). However, unlike most true HR, it takes about 5 days rather than hours to develop. Furthermore, the virus was able move systemically, albeit at a very low level.

Inheritance of the TYLCV resistance in the progeny of a LAB x QLD cross, showed that the resistance is likely to be from a single dominant allele present in the QLD background. Given the well-known RDRγ type Begomovirus resistance genes in tomato and pepper (Koeda et al., 2022), and that overexpressing the *Ty-1* gene in *N. benthamiana* LAB has been shown to confer strong resistance to TYLCV infection (Voorburg et al., 2020), the RDRγ type NbRDR3 and/or NbRDR5 genes in QLD seemed the likely source(s) of TYLCV resistance. However, when these genes were dysfunctionalised in QLD by CRISPR-Cas9 mediated deletions, either separately or together, the mutated plants remained highly resistant to TYLCV. Furthermore, the resistance appeared to be virus specific in that QLD was severely affected by TYDV, whereas an RDR3-mediated resistance, likely operating by small RNAs and DNA methylation (Butterbach et al., 2014), might be expected to confer generic resistance against a range of DNA viruses.

With the preclusion of RDR3/5 as the source of the TYLCV resistance in QLD, two other possibilities were examined, an NLR-like gene and a Pelota-like gene. However, we found strong evidence that the resistance in QLD is not operating through either the classic hypersensitive response of an NLR gene or in a Pelota-like homozygous recessive gene mechanism. Interestingly, we have identified an insertion in the 5’ end of the NbQPelota b gene that completely removes the N-terminus of the protein. A recent study by Ren et al. (2022) has shown that silencing of Pelota genes in naturally susceptible *N. benthamiana* (LAB) confers resistance against several geminiviruses including TYLCV. One scenario to explain this result and the resistance inherited from QLD could be that NbQPelota b exerts a dominant negative effect silencing the functional forms of NbQPelota and NbLPelota. To test this, we genotyped a population from a selfed LAB x QLD cross (F1S1) that showed segregation for TYLCV resistance. This analysis confirmed that there is no correlation between inheritance of the NbQPelota b allele and the resistant phenotype. Altogether, this study suggests that QLD possesses an undiscovered, novel and effective single resistance gene against TYLCV, which has the potential to be utilised in the field. The remaining challenge is to identify this gene. With the recently available chromosome-level, reference quality genome sequences of both LAB and QLD (www.apollo.nbenth.com; Ranawaka et al., 2023), this seems highly possible through a genome wide association study (GWAS) approach (Schultink et al., 2019).

## Data availability statement

All relevant data are presented in the article and **Supplementary Material**, further inquiries can be directed to SH, https://satomi.hayashi@qut.edu.au.

## Author contributions

SH: Conceptualization, Data curation, Formal analysis, Investigation, Methodology, Supervision, Validation, Writing – original draft, Writing – review & editing. JS: Data curation, Investigation, Writing – review & editing. JB: Supervision, Writing – review & editing. FdF: Supervision, Writing – review & editing. PW: Conceptualization, Funding acquisition, Project administration, Resources, Supervision, Validation, Writing – review & editing.

## Supplementary material

Supplementary Figure 1 Genotype PCR of 24 LAB x QLD F1S1 individuals for resistant gene candidate. **(A)** RDR1: with product size 275 bp and 347 bp for LAB and QLD allele, respectively. **(B)** RDR3: A full length PCR product of 778 bp for QLD allele, and 269 bp and 511 bp DNA fragments for LAB allele after digestion with *Hpa*II. **(C)** Pelota b: A full length PCR product of 750 bp for LAB allele, and 368 bp and 388 bp DNA fragments for QLD after digestion with *Scr*FI. **(D)** Present/absence PCR analysis for the 5’ insertion in NbQPelota b allele with expected product size of 676 bp.
