# Supplementary figures and images for: Exploring the source of TYLCV resistance in Nicotiana benthamiana
Source: Front Plant Sci. 2024 May 28;15:1404160. doi: 10.3389/fpls.2024.1404160 (PMC11165019; doi:10.3389/fpls.2024.1404160)

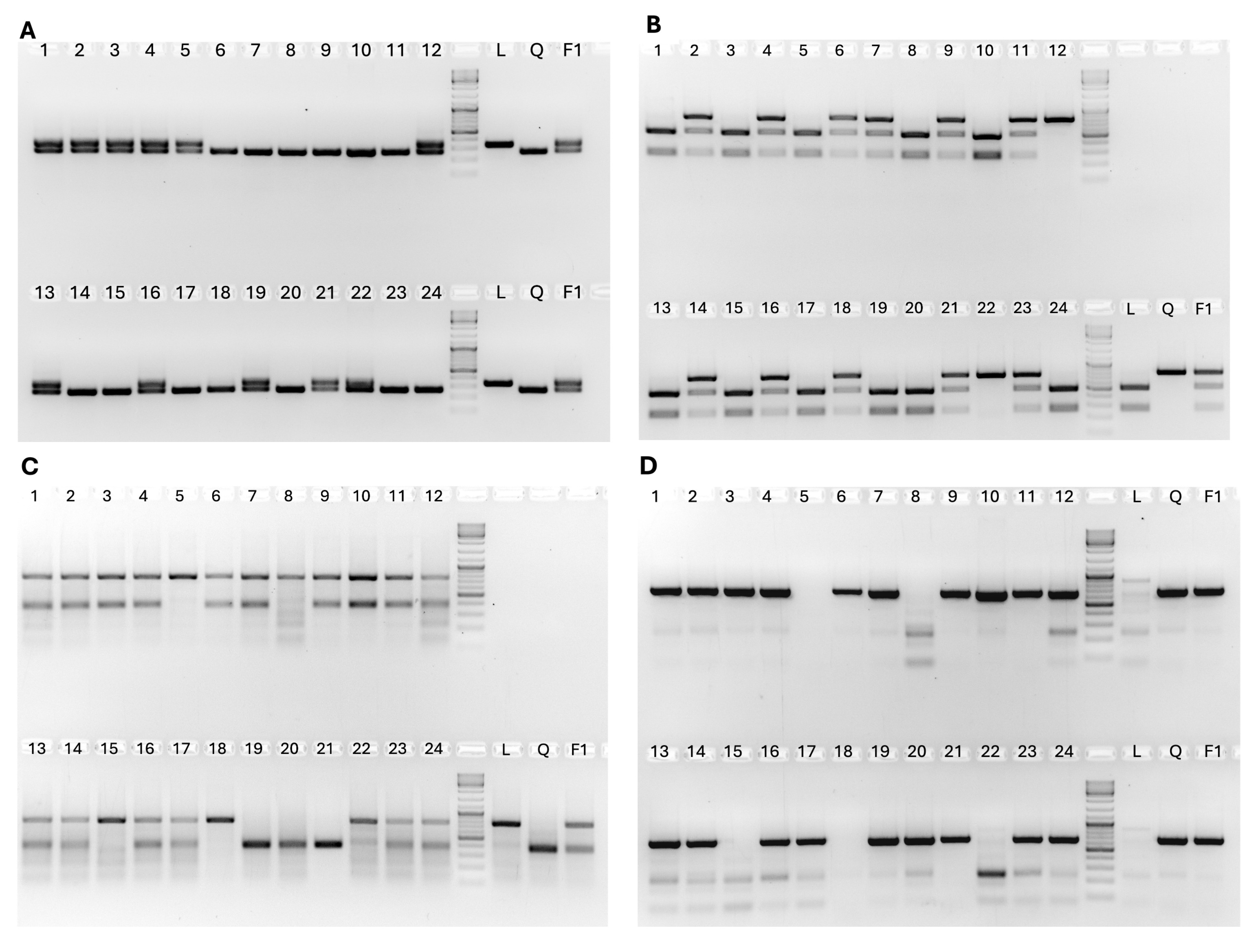

Supplement: Supplementary Figure 1 — Genotype PCR of 24 LAB x QLD F1S1 individuals for resistant gene candidate. (A) RDR1: with product size 275 bp and 347 bp for LAB and QLD allele, respectively. (B) RDR3: A full length PCR product of 778 bp for QLD allele, and 269 bp and 511 bp DNA fragments for LAB allele after digestion with HpaII. (C) Pelota b: A full length PCR product of 750 bp for LAB allele, and 368 bp and 388 bp DNA fragments for QLD after digestion with ScrFI. (D) Present/absence PCR analysis for the 5’ insertion in NbQPelota b allele with expected product size of 676 bp. [file Image_1.jpeg]
